# Microwave Device-Assisted Pericardiectomy for Immunoglobulin G4–Related Constrictive Pericarditis: A Case Report

**DOI:** 10.70352/scrj.cr.26-0154

**Published:** 2026-06-26

**Authors:** Yuriko Kiriya, Keiichi Ishiwari, Tomomitsu Takagi, Kay Maeda, Yoko Matsumura, Michio Yoshitake, Kenji Kobayashi, Takashi Kunihara

**Affiliations:** 1 Department of Cardiac Surgery, The Jikei University School of Medicine, Tokyo, Japan; 2 Department of Pathology, The Jikei University School of Medicine, Tokyo, Japan

**Keywords:** microwave device, pericardiectomy, Immunoglobulin G4-related disease, constrictive pericarditis

## Abstract

**INTRODUCTION:**

Immunoglobulin G4 (IgG4)-related constrictive pericarditis (CP) is a rare condition with no standardized treatment. Surgical pericardiectomy is challenging due to dense adhesions and the risk of injury to the phrenic nerves and heart. Conventional energy devices carry risks of thermal damage. We report a novel application of a microwave ablation device (Acrosurg; Nikkiso, Tokyo, Japan) for radical pericardiectomy, highlighting its advantages in preventing nerve injury and ensuring hemostatic control.

**CASE PRESENTATION:**

A 79-year-old male patient with recurrent heart failure due to IgG4-related CP underwent off-pump radical pericardiectomy using a microwave ablation device (Acrosurg) and a positioner. The Acrosurg device enabled extensive and precise resection up to 1.5 cm from the phrenic nerves, achieving near total pericardiectomy without thermal injury, arrhythmia, or nerve paralysis. Postoperatively, cardiac index increased and central venous pressure normalized. The patient remained recurrence-free without steroids.

**CONCLUSIONS:**

This case demonstrated that the Acrosurg device allows safe and curative resection for CP, highlighting its utility as an innovative surgical tool.

## Abbreviations

CI
: cardiac index
CP
: constrictive pericarditis
CPB
: cardiopulmonary bypass
IgG4-RD
: immunoglobulin G4-related disease
IVC
: inferior vena cava
LVEF
: left ventricular ejection fraction
PWCP
: pulmonary capillary wedge pressure

## INTRODUCTION

IgG4-RD is a systemic disorder characterized by lymphoplasmacytic infiltration rich in IgG4-positive plasma cells, storiform fibrosis, and often elevated serum IgG4 concentration.^1)^ While it can affect various organs, cardiac involvement, particularly IgG4-related CP, is very rare, with only a few cases reported in the literature.^2,3)^ Standardized treatment strategies have yet to be established but may include surgery and/or corticosteroid therapy.^2,4)^

However, surgical procedures for CP remain technically challenging due to dense adhesions, risk of hemorrhage, potential for the induction of arrhythmia, risk of coronary artery injury, and proximity to vital structures, particularly the phrenic nerves.

Conventional methods using energy devices such as electrocautery carry risks of thermal injury to neural tissues and coronary arteries, as well as the potential to induce arrhythmia. Here, we report a novel application of a microwave ablation device (Acrosurg; Nikkiso, Tokyo, Japan) for radical pericardiectomy, highlighting its advantages in preventing phrenic nerve injury and providing hemostatic control.

## CASE PRESENTATION

A 79-year-old man with a history of hypertension, diabetes mellitus, hyperlipidemia, left hemiparesis following right brainstem infarction, and pancreaticoduodenectomy for bile duct cancer presented with exertional dyspnea and palpitations 4 years previously, leading to a diagnosis of congestive heart failure. Despite optimal medical management, he was hospitalized repeatedly. Further cardiological investigation confirmed CP, and he was referred to our department for surgical treatment.

**Fig. 1:**
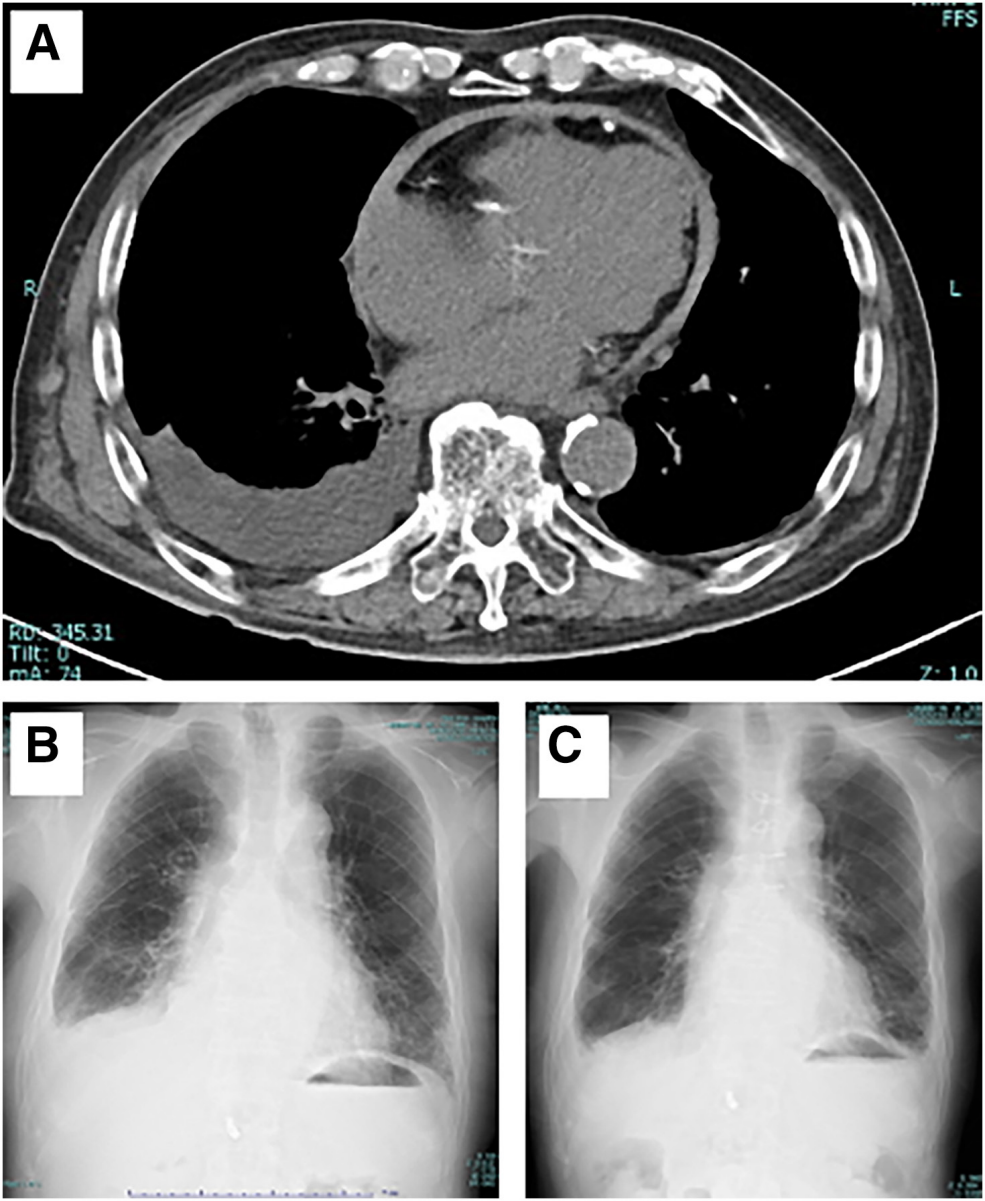
Preoperative imaging findings. (**A**) Preoperative CT image showing diffuse pericardial thickening. (**B**) Preoperative chest radiograph demonstrating cardiomegaly and clear lung fields. (**C**) Postoperative chest radiograph showing a reduced cardiac silhouette and no evidence of diaphragmatic elevation, suggesting absence of phrenic nerve injury.

**Fig. 2:**
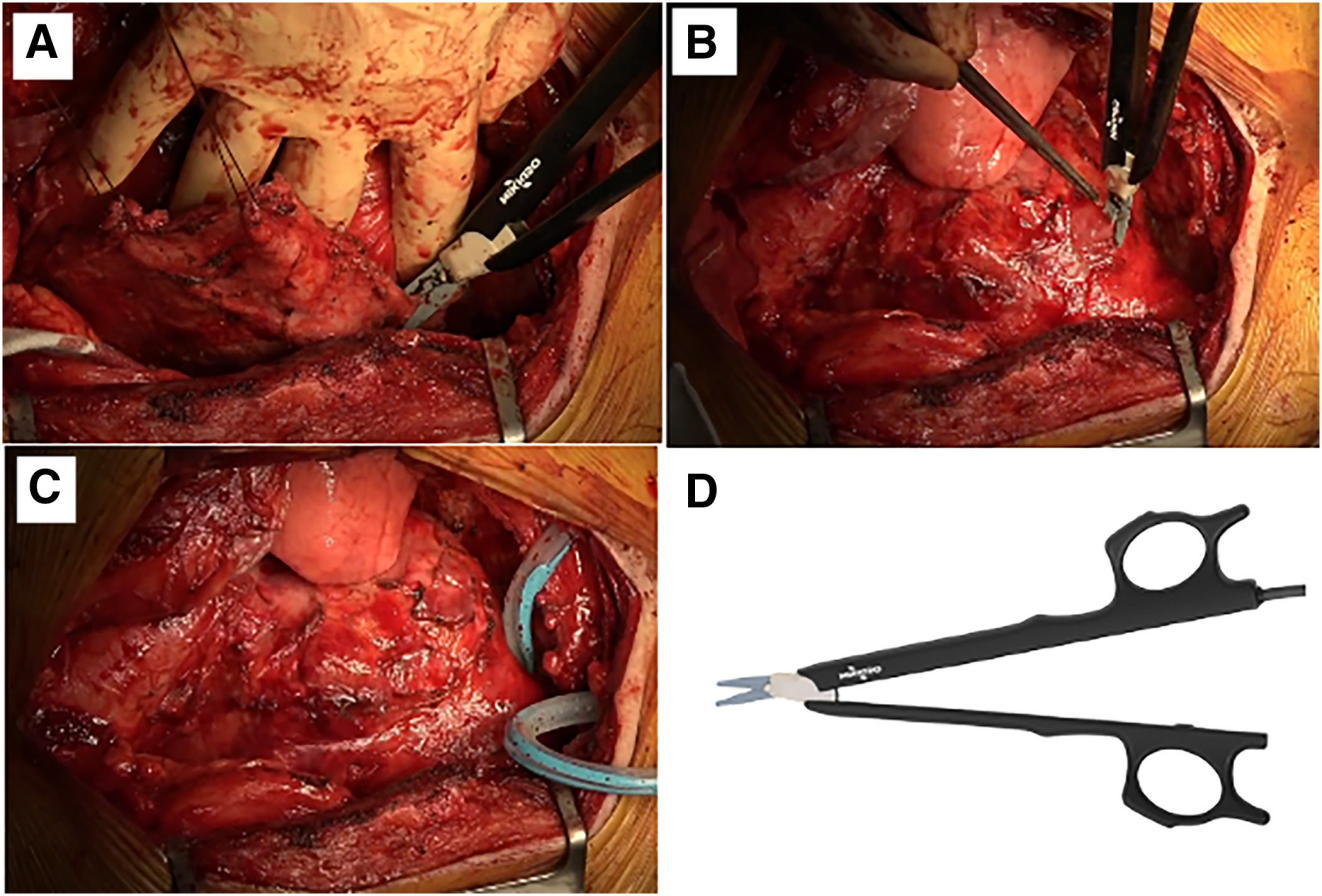
(**A**, **B**) The microwave device offered controlled, localized energy delivery, which may reduce the risk of stimulating arrhythmia and damaging adjacent neural structures such as the phrenic nerve. (**C**) Pericardiectomy, pericardiotomy, and epicardial crosshatching incision using the microwave device. (**D**) Effective use of a microwave ablation device for pericardial resection.

Preoperative examination revealed pulsus paradoxus and Kussmaul’s sign. Blood tests showed elevated B-type natriuretic peptide (241.6 pg/mL) and IgG4 (244.1 mg/dL). CT demonstrated significant diffuse pericardial thickening (8 mm) without calcification (**Fig. 1**). Echocardiography revealed pericardial adhesion, septal bounce, and preserved left ventricular function with LVEF of 66.6%. Cardiac catheterization confirmed hemodynamic features of constriction: dip-and-plateau pattern, elevated and equalized diastolic pressures (right ventricular end-diastolic/systolic pressure 21/29 mmHg; right atrial pressure/PCWP: 23/29 mmHg; right/left ventricular end diastolic pressure difference: 3 mmHg), and reduced CI (1.45 L/min/m^2^). The patient underwent pericardiectomy without CPB. The pericardium was diffusely thickened and adherent. Using the microwave ablation device (Acrosurg), the thickened parietal pericardium was widely resected up to 1.5 cm anterior to both phrenic nerves using a positioner (Urchin Evo; Medtronic, Minneapolis, MN, USA) to avoid thermal injury. A waffle procedure (cross-hatching incision) was also performed on the epicardium over the anterior right ventricle (**Fig. 2** and **Supplementary material: Video S1**). The total operative time was 3 h 25 min, with estimated blood loss of 1050 mL. The procedure was completed without the need for blood transfusion.

The postoperative course was uneventful. The patient was extubated on POD 1 and transferred from the ICU on POD 2. Chest radiography showed no phrenic nerve paralysis (**Fig. 1B** and **1C**). Hemodynamics improved significantly (CI: 2.8 L/min/m^2^) and postoperative echocardiography showed an LVEF of 60.8%, which was mildly decreased from the preoperative value, consistent with relief of ventricular constriction. Postoperative echocardiography further demonstrated resolution of the septal bounce, normal respiratory variation of mitral inflow (ratio of early to late diastolic filling velocities = 1.1, and ratio of early transmitral inflow velocity to mitral annular early diastolic velocity = 8), and a reduction in IVC diameter with preserved respiratory collapsibility, confirming successful relief of diastolic constriction. The patient was discharged on POD 19.

**Fig. 3:**
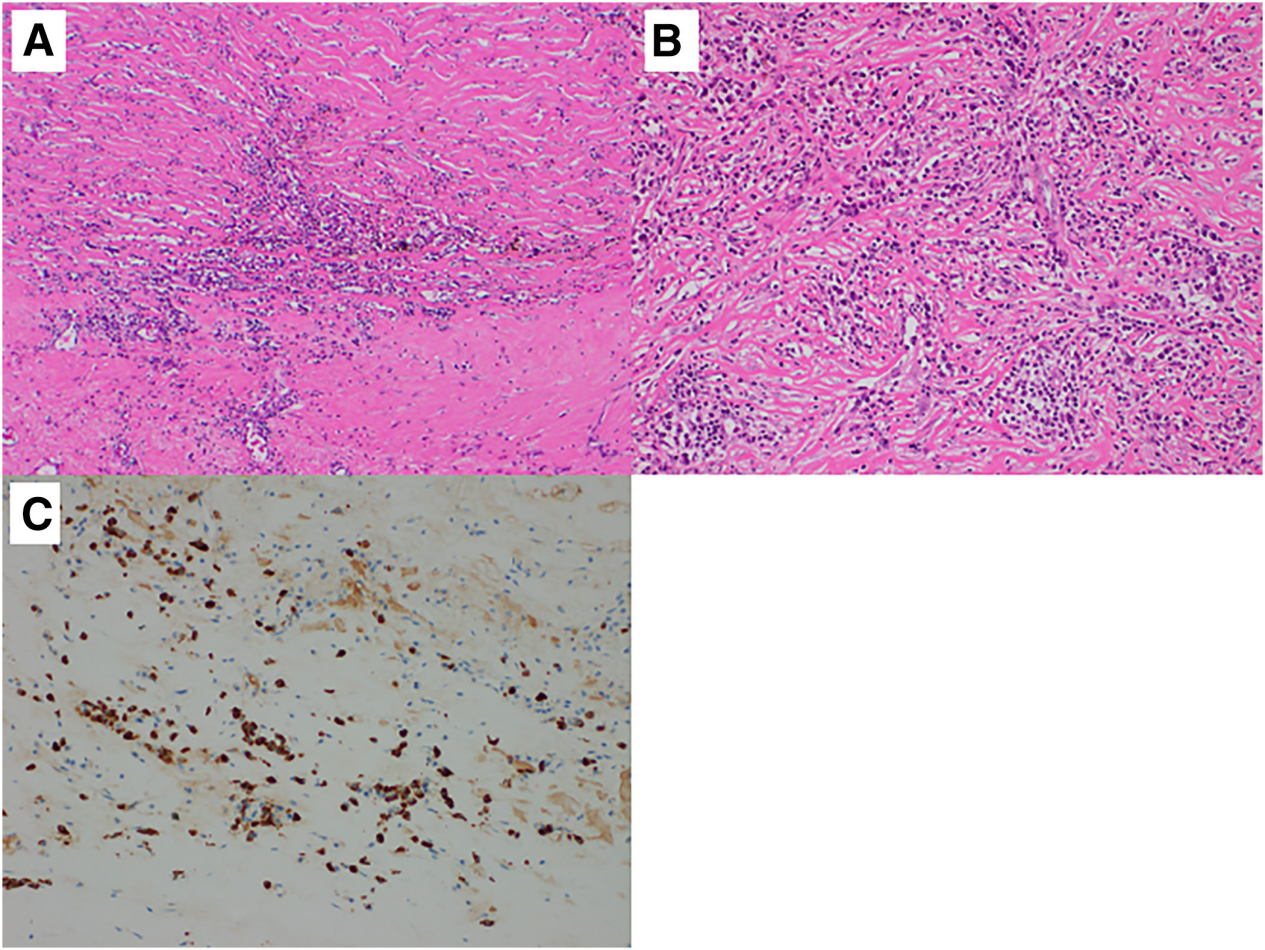
Photomicrographs of the resected pericardium. (**A**) Hematoxylin and eosin staining showing fibrosis and chronic inflammation. (**B**) Storiform fibrosis increased the probability of IgG4-RD. (**C**) Immunostaining revealed numerous IgG4-positive plasma cells (IgG4/IgG ratio: 66%). IgG, immunoglobulin G; IgG4-RD, immunoglobulin G4-related disease

Pathological examination of the resected pericardium revealed fibrotic tissue with lymphoplasmacytic infiltration. Immunostaining showed a high ratio of IgG4-positive plasma cells (IgG4/IgG ratio: 66%) (**Fig. 3**), confirming the diagnosis of IgG4-RD. At 5-month follow-up, the patient remained asymptomatic without steroid therapy and with a stable serum IgG4 level of 260 mg/dL.

## DISCUSSION

We encountered a rare case of IgG4-related CP, which highlighted the utility of the Acrosurg microwave ablation device for safe pericardiectomy and the potential for surgical resection alone to induce remission in selected cases.

IgG4-related CP presents a significant surgical challenge due to severe, diffuse fibrous adhesions between the thickened pericardium and the epicardium.^2,4)^ Traditional dissection tools such as electrocautery and ultrasonic scalpels carry risks of arrhythmia and phrenic nerve injury due to thermal spread or energy transmission.^4)^

The microwave device used here (Acrosurg) operates by agitating water molecules within the tissue to generate heat locally for cutting and coagulation. Unlike conventional devices, the Acrosurg does not require switching between cut and coagulation modes; it mechanically cuts with scissor-shaped tips while simultaneously coagulating with microwave energy. The Acrosurg does not have adjustable power settings in the same manner as conventional electrosurgery; the output is standardized for its scissors-type handpiece.

According to manufacturer specifications (Nikkiso), the lateral thermal spread is limited to approximately 1.0–1.5 mm in *ex vivo* settings, which is narrower than that reported for conventional ultrasonic scalpels (typically 1.5–3.0 mm).^5)^ This reduced lateral thermal dispersion likely contributes to a lower risk of phrenic nerve injury and arrhythmia induction compared to traditional energy devices.

Furthermore, avoiding CPB is desirable, as the use of CPB during pericardiectomy may increase the risk of bleeding and other complications. In the present case, off-pump resection was successfully achieved with this device, and the postoperative course was uneventful.

The optimal postoperative management for IgG4-RD remains a matter of some debate. While some cases require adjuvant steroid therapy to control systemic inflammation or prevent recurrence,^2,4)^ others—like the present case—have been reported to remain recurrence-free with surgical management alone over the long term.^3)^ The definitive histopathological diagnosis is crucial and relies on the presence of dense lymphoplasmacytic infiltrates, a large number of IgG4-positive plasma cells (>10–30/high-power field), an elevated IgG4/IgG ratio (>40%), and characteristic fibrotic patterns (storiform fibrosis, obliterative phlebitis).^1,2,4)^ The high IgG4/IgG ratio (66%) in our patient strongly supported the diagnosis. This report has several limitations. First, it describes a single case, and the generalizability of the findings is therefore limited. Second, comprehensive echocardiographic assessment of diastolic function before and after surgery was not systematically performed; parameters such as left ventricular end-diastolic volume, respiratory variation of mitral inflow, and IVC dynamics were not recorded. Future cases should incorporate standardized diastolic assessment to objectively document the efficacy of this procedure.

## CONCLUSIONS

In conclusion, pericardiectomy using a microwave dissection device can be safe and effective for the treatment of IgG4-related CP, allowing extensive resection without CPB and minimizing the risk of thermal nerve injury. Surgery alone may induce lasting remission in a subset of patients, avoiding the need for long-term steroid therapy.

## SUPPLEMENTARY MATERIALS

Supplementary Video **Video S1** Surgical view of microwave device (Acrosurg)-assisted off-pump pericardiectomy for immunoglobulin G4-related constrictive pericarditis.

## DECLARATIONS

### Availability of data and materials

The data that support the findings of this study are available from the corresponding author upon reasonable request.
